# Correlation of serum vitamin D, calcium-phosphorus metabolism and functional outcome in osteoporotic fractures

**DOI:** 10.6026/973206300223375

**Published:** 2026-06-30

**Authors:** Sonendra Kumar Sharma, Sandeep Kumar Jain, Niket Raj Garg, Vishnu Kumar gupta, Rajesh Ahirwar, Parul Nema

**Affiliations:** 1 Department of Orthopaedics surgery, SRVS Medical College, Shivpuri, Madhya Pradesh, India; 2 Department of Medicine, Chhindwara Institute of Medical sciences, Madhya Pradesh, India; 3 Department of Orthopaedics, Baba kinaram Autonomous State Medical College, Chandauli, Uttar Pradesh, India; 4 Department of Community Medicine, SRVS Medical College, Shivpuri, Madhya Pradesh, India; 5 Department of Pathology, SRVS Medical College, Shivpuri, Madhya Pradesh, India

**Keywords:** Vitamin D, 25-hydroxyvitamin D (25-OH-D), calcium, phosphorus, osteoporotic fracture, functional outcome, Barthel Index (BI), hip fracture

## Abstract

Osteoporotic fractures are associated with poor functional recovery in older adults. Therefore, it is of interest to evaluate serum 25-OH-D, calcium, phosphorus, and PTH in 120 fragility fracture patients. Hence, Vitamin D deficiency was observed in 65% of patients and showed significant positive correlation with 6-month functional recovery scores. Elevated PTH levels were negatively associated with recovery outcomes, while calcium and phosphorus were not significant predictors. Thus, we report serum vitamin D as an independent predictor of long-term functional recovery after osteoporotic fractures.

## Background:

Osteoporotic fractures represent a major cause of morbidity, disability and loss of independence among older adults worldwide. As the global population ages, the incidence of osteoporosis and its associated fractures continues to rise, leading to a substantial healthcare burden [1]. Osteoporosis is characterized by reduced bone mass and deterioration of bone tissue, which makes bones more fragile and susceptible to fractures, even from minimal trauma. The most common fractures in older adults are hip, spine and wrist fractures, which often result in significant long-term disability, reduced quality of life and sometimes even death [2]. Beyond the physical consequences of these fractures, there are also psychological and social impacts, as individuals may experience depression, anxiety and a loss of social engagement due to physical limitations [3]. Vitamin D plays a crucial role in the regulation of calcium and phosphorus homeostasis in the body, which is essential for maintaining bone health and preventing fractures [4]. In addition to its role in bone mineralization, vitamin D is also important for muscle strength and fall prevention, which are critical in reducing the risk of fractures. Vitamin D enhances the absorption of calcium in the intestines and helps in maintaining adequate levels of calcium and phosphorus in the blood, both of which are essential for bone strength [5]. Furthermore, vitamin D has a direct effect on muscle function and its deficiency is associated with muscle weakness and an increased risk of falls. This is particularly important in older adults, as muscle strength and balance are key factors in preventing falls and fractures [6]. There is growing evidence that vitamin D deficiency is commonly observed in individuals with fragility fractures. Studies have shown that individuals who suffer from such fractures frequently have insufficient levels of vitamin D, which may contribute to poor bone quality and increase the likelihood of fractures [7]. Moreover, vitamin D deficiency may also impair fracture healing and negatively affect the outcomes following surgery, making it a significant concern for healthcare providers managing osteoporotic fractures in older patients [8]. Despite the observed association between vitamin D deficiency and fragility fractures, the exact role of vitamin D supplementation in fracture healing and recovery remains unclear. Randomized controlled trials investigating the effects of vitamin D supplementation on fracture healing have produced inconsistent results [9]. While some studies suggest that vitamin D supplementation may improve bone healing and functional recovery, others have failed to demonstrate significant benefits. The disparity in findings may be due to differences in study design, sample size, or the timing and dose of vitamin D supplementation, among other factors. Additionally, other markers of mineral metabolism, such as calcium, phosphate and parathyroid hormone (PTH), may also play a role in fracture healing and recovery. The relative importance of these markers, in comparison to vitamin D, is still not well understood [10, 11 and 12]. Therefore, it is of interest to assess the associations between functional recovery at three and six months following osteoporotic fractures in older persons, baseline serum 25-OH-D and calcium-phosphorus metabolism (including PTH).

## Methods:

## Study design and setting:

This Prospective observational study was carried out in a tertiary care teaching hospital, Central India from January 2023 to December 2024 (02 year duration). The institutional ethics committee approved the study and all participants (or surrogates) provided written informed consent.

## Inclusion criteria:

[1] Patients age ≥ 60 years, with both genders

[2] Low-energy fragility fracture (hip-femoral neck/intertrochanteric, distal radius, proximal humerus)

[3] Admitted for management within 7 days of injury

## Exclusion criteria:

[1] High energy trauma

[2] Pathologic fractures (metastasis) or active malignancy

[3] Chronic kidney disease stage IV-V or primary hyperparathyroidism,

[4] Current high-dose vitamin D therapy (>2000 IU/day) for >1 month prior 

## Data collection:

Baseline demographics, comorbidities (Charlson comorbidity index), fracture type, pre-injury functional status (retrospective Barthel Index) and in-hospital complications were recorded. Blood samples within 48 hours of admission were obtained for:

[1] Serum 25-hydroxyvitamin D (chemiluminescent immunoassay), reported in ng/mL.

[2] Serum total calcium, albumin, inorganic phosphate, parathyroid hormone (PTH) and Creatinine (to estimate eGFR).

[3] Bone mineral density (dual-energy x-ray absorptiometry).

[4] Vitamin D status categories: deficiency < 20 ng/mL, insufficiency 20-29 ng/mL, sufficient ≥ 30 ng/mL (commonly used clinical thresholds).

## Outcomes:

[1] Primary outcome: functional recovery at 6 months measured by Barthel Index (BI) for all patients; Hip fractures also assessed with Harris Hip Score (HHS).

[2] Secondary outcomes: BI at 3 months, time to independent ambulation, radiographic union for non-hip extremity fractures (where applicable) and complication rates.

## Follow-up:

Assessments performed at discharge, 3 months and 6 months. Missing data minimized by telephone reminders; loss to follow up recorded.

## Sample size:

We planned for n = 120 based on pragmatic recruitment (power calculation for detecting a moderate correlation r = 0.30 between 25-OH-D and BI at 6 months: α = 0.05, β = 0.80 requires ≈85; we enrolled 120 to allow subgroup analyses and 20% attrition).

## Statistical analysis:

Statistical analyses were performed using SPSS version 26. Continuous variables are mean ± SD or median (IQR) as appropriate. Categorical variables as counts and percentages. Spearman correlation tested relationships between biochemical markers and functional scores. Comparisons between vitamin D groups used ANOVA and chi-square tests. Multivariable linear regression was done. A p-value < 0.05 considered significant.

## Results:

A total of 120 patients with fragility fractures were enrolled in the study, of whom 112 patients (93.3%) successfully completed the 6-month follow-up assessment. The baseline demographic and clinical characteristics of the study population are summarized in Table 1 The mean age of the participants was 74.1 ± 7.9 years and females constituted 68.3% of the cohort. Hip fractures were the most common fracture type, followed by distal radius and proximal humerus fractures. The median pre-injury Barthel Index (BI) was 95, indicating relatively preserved baseline functional status, while the median Charlson comorbidity index was 2. The mean estimated glomerular filtration rate (eGFR) was 64.5 ± 18.1 mL/min/1.73 m^2^ (Table 1). Baseline biochemical parameters demonstrated a high prevalence of vitamin D deficiency among the participants. The mean serum 25-hydroxyvitamin D (25-OH-D) level was 16.2 ± 7.9 ng/mL, with 65.0% of patients categorized as vitamin D deficient, 20.0% as insufficient and only 15.0% as sufficient. The mean corrected serum calcium and phosphate levels were 8.6 ± 0.5 mg/dL and 3.5 ± 0.6 mg/dL, respectively. The median serum parathyroid hormone (PTH) concentration was 58 pg/mL with an interquartile range of 42-76 pg/mL (Table 2). Functional recovery progressively improved during follow-up. The mean Barthel Index score increased from 62.7 ± 18.9 at 3 months to 76.8 ± 15.3 at 6 months after fracture. Among the hip fracture subgroup (n = 64), the mean Harris Hip Score (HHS) at 6 months was 76.3 ± 14.6, indicating moderate functional recovery. Correlation analysis revealed a significant positive association between baseline serum 25-OH-D levels and 6-month Barthel Index scores (r = 0.42, p < 0.001). In contrast, serum PTH levels demonstrated a significant negative correlation with functional recovery outcomes (r = -0.31, p = 0.001). Serum calcium and phosphate levels did not show statistically significant correlations with 6-month BI scores (Table 3). Multivariable linear regression analysis was performed to identify independent predictors of 6-month functional recovery after adjusting for age, sex, baseline BI, Charlson comorbidity index, fracture type and eGFR. Higher serum 25-OH-D levels remained an independent positive predictor of improved 6-month Barthel Index scores (β = 0.34, 95% CI: +0.12 to +0.56, p = 0.002). Elevated serum PTH levels independently predicted poorer functional recovery (β = -0.19, 95% CI: -0.33 to -0.05, p = 0.01). Baseline BI also showed a strong positive association with recovery outcomes, whereas age and fracture type were not statistically significant predictors in the adjusted model (Table 4).

## Discussion:

Vitamin D deficiency was prevalent (65%) in this prospective sample of older persons with osteoporotic fractures. After controlling for covariates, lower baseline blood 25-OH-D remained an independent predictor of 6-month functional status and marginally linked with worse functional recovery at 3 and 6 months. A worse result was also independently linked to elevated PTH, a sign of secondary hyperparathyroidism. On the other hand, baseline measurements of serum total calcium and inorganic phosphate showed weak, non-significant correlations with functional recovery following correction. Although the research is inconsistent, a number of observational studies and reviews have found a link between vitamin D insufficiency and lower functional results, greater rates of complications and impaired fracture healing. Although the evidence was conflicting and further research was required, Yu *et al.* (2017) [13] came to the conclusion that adult fracture healing may be impacted by baseline vitamin D levels. Vitamin D deficiency is common among hip fracture patients, according to meta-analyses and more recent cohort work and may have a detrimental impact on recovery and quality of life after the fracture. However, interventional trials using vitamin D supplementation alone have yielded conflicting results for enhancing healing and function. Our finding of a link with functional recovery is supported by a 2024 meta-analysis by Llombart *et al.* [14] that suggested vitamin D insufficiency may have a detrimental impact on physical function and quality of life following hip fracture. The authors did, however, draw attention to study heterogeneity. Vitamin D controls bone mineralization, calcium and phosphate absorption, muscular function and balance all processes important to mobility and fracture repair. When combined with a vitamin D shortage, secondary hyperparathyroidism may hasten bone resorption and impair muscular function, which could lead to a less successful functional recovery. Systemic nutrition, metabolic state and mineral availability all affect fracture healing. According to our research, systematic screening for vitamin Ddeficiency may reveal a modifiable risk factor linked to recovery in older persons who present with fragility fractures. Despite conflicting results from studies on vitamin D supplementation for fracture healing, several recommendations advocate for addressing proven deficiencies, especially in light of the benefits for muscle and fall risk in older persons [15]. Supplementation decisions should be made on an individual basis and more randomized studies with functional recovery objectives are necessary.

## Strengths and limitations:

Strengths include multivariable correction, standardized functional evaluations at three and six months and prospective design. Single-center, moderate sample size, observational design (cannot establish causality); possible residual confounding (e.g., nutritional status, intensity of rehabilitation) and absence of randomized supplements are the limitations. Single baseline biochemical measurements were employed; dynamic changes and post-discharge adherence to any supplementation were not monitored.

## Conclusion:

Vitamin D deficiency was prevalent and was linked to a lower functional recovery at six months. After normalization, baseline measurements of calcium and phosphate were not independently predictive in this group of older persons with osteoporotic fractures. These observational data give justification for interventional trials examining whether correcting vitamin D insufficiency enhances functional recovery and support routine evaluation of vitamin D status following fragility fractures.

## Figures and Tables

**Table 1:** Baseline characteristics (n = 120)

**Variable**	**Value**
Age, mean ±SD (years)	74.1 ±7.9
Female, n (%)	82 (68.3)
Fracture type, n (%)	Hip 64 (53.3); Distal radius 32 (26.7);
	Proximal humerus 24 (20.0)
Pre-injury BI (median, IQR)	95 (90-100)
Charlson index, median (IQR)	2 (1-3)
eGFR (mL/min/1.73 m^2^), mean ±SD	64.5 ±18.1

**Table 2:** Baseline biochemical markers

**Marker**	**Mean ±SD or median (IQR)**
25-OH-D (ng/mL)	16.2 ±7.9
Corrected calcium (mg/dL)	8.6 ±0.5
Phosphate (mg/dL)	3.5 ±0.6
PTH (pg/mL)	58 (42-76)

**Table 3:** Correlation coefficients between baseline biochemical markers and 6-month Barthel Index (n=112)

**Marker**	**Spearman r**	**p-value**
25-OH-D (ng/mL)	0.42	<0.001
Corrected calcium (mg/dL)	0.12	0.18
Phosphate (mg/dL)	-0.05	0.56
PTH (pg/mL)	-0.31	0.001

**Table 4:** Multivariable linear regression predicting 6-month barthel index

**Predictor**	**β (standardized)**	**95% CI**	**p**
25-OH-D (per 10 ng/mL)	0.34	+0.12 to +0.56	0.002
PTH (per 10 pg/mL)	-0.19	-0.33 to -0.05	0.01
Age (per 5 years)	-0.08	-0.25 to +0.09	0.34
Baseline BI	0.39	+0.21 to +0.57	<0.001
Fracture type (hip vs other)	-0.06	-0.23 to +0.11	0.47
